# Automated Identification of Acute Hepatitis B Using Electronic Medical Record Data to Facilitate Public Health Surveillance

**DOI:** 10.1371/journal.pone.0002626

**Published:** 2008-07-09

**Authors:** Michael Klompas, Gillian Haney, Daniel Church, Ross Lazarus, Xuanlin Hou, Richard Platt

**Affiliations:** 1 Department of Ambulatory Care and Prevention, Harvard Medical School and Harvard Pilgrim Health Care, Boston, Massachusetts, United States of America; 2 Channing Laboratory, Department of Medicine, Brigham and Women's Hospital, Harvard Medical School, Boston, Massachusetts, United States of America; 3 Massachusetts Department of Public Health, Boston, Massachusetts, United States of America; Health Protection Agency, United Kingdom

## Abstract

**Background:**

Automatic identification of notifiable diseases from electronic medical records can potentially improve the timeliness and completeness of public health surveillance. We describe the development and implementation of an algorithm for prospective surveillance of patients with acute hepatitis B using electronic medical record data.

**Methods:**

Initial algorithms were created by adapting Centers for Disease Control and Prevention diagnostic criteria for acute hepatitis B into electronic terms. The algorithms were tested by applying them to ambulatory electronic medical record data spanning 1990 to May 2006. A physician reviewer classified each case identified as acute or chronic infection. Additional criteria were added to algorithms in serial fashion to improve accuracy. The best algorithm was validated by applying it to prospective electronic medical record data from June 2006 through April 2008. Completeness of case capture was assessed by comparison with state health department records.

**Findings:**

A final algorithm including a positive hepatitis B specific test, elevated transaminases and bilirubin, absence of prior positive hepatitis B tests, and absence of an ICD9 code for chronic hepatitis B identified 112/113 patients with acute hepatitis B (sensitivity 97.4%, 95% confidence interval 94–100%; specificity 93.8%, 95% confidence interval 87–100%). Application of this algorithm to prospective electronic medical record data identified 8 cases without false positives. These included 4 patients that had not been reported to the health department. There were no known cases of acute hepatitis B missed by the algorithm.

**Conclusions:**

An algorithm using codified electronic medical record data can reliably detect acute hepatitis B. The completeness of public health surveillance may be improved by automatically identifying notifiable diseases from electronic medical record data.

## Introduction

Public health surveillance for notifiable diseases has traditionally relied upon clinicians to spontaneously report new diagnoses of relevant conditions. Clinician-initiated reporting, however, is often incomplete and delayed.[1], [2] Electronic laboratory reporting systems have improved both the volume and timeliness of reporting [3], [4], [5], [6] but these systems have important limitations: they cannot report purely clinical diagnoses (such as culture-negative tuberculosis), indicate when a result is likely a false positive (such as positive hepatitis A IgM in an asymptomatic patient getting screening tests), nor render diagnoses that require integration of laboratory tests along with patient clinical data and prior test results (such as acute hepatitis B). The lack of specificity in electronic laboratory reporting increases workload for health departments compelled to investigate suggestive but non-specific lab results.[7] In addition, electronic laboratory reporting systems do not report clinical data that can be crucial to guiding public health interventions such as patients' pregnancy status, prescribed treatments, and full contact information.

Electronic medical record systems are a promising new strategy to improve public health surveillance.[8] These systems encode a wide array of clinical data including patient demographics, current and prior diagnoses, medication prescriptions, and laboratory results. These data might potentially be used to detect notifiable diseases that cannot be found by electronic laboratory reporting systems as well as to convey important information to public health authorities on patient demographics, clinical status, and prescribed treatments. Accurate identification of complex diagnoses from electronic medical records, however, requires the development of novel detection algorithms since diagnostic codes alone, such as International Classification of Diseases Ninth Revision codes (ICD9), are imprecise.[9], [10]

In order to assess the feasibility of public health surveillance for complex notifiable diseases using electronic medical record data, we sought to develop an algorithm to identify cases of acute hepatitis B using electronic medical record data. Acute hepatitis B was chosen as a “proof of principle” condition because it is a complex diagnosis of public health importance that is largely transparent to electronic laboratory reporting systems.

Accurate identification of acute hepatitis B is essential to public health practice. Public health practitioners seek acute cases to gauge the changing epidemiology of hepatitis B and the impact of universal vaccination programs.[11] Acute cases also trigger high-priority interventions to limit the spread of disease. Clinician-initiated reporting of acute hepatitis B, however, is typically incomplete, delayed, and inaccurate: public health departments have found that up to 40% of cases reported by clinicians as acute hepatitis B turn out to be chronic infection upon further investigation.[12] Electronic laboratory reporting systems have improved both the volume and timeliness of hepatitis B case reports but these systems typically only report the presence of a positive test for hepatitis B–they cannot distinguish between acute and chronic infections.[6]

The central challenge for both clinicians and lab surveillance systems in identifying acute hepatitis B is distinguishing acute cases from “flares” of previously undiagnosed chronic disease.[11], [12] Both can present with markedly elevated transaminases and positive hepatitis B specific tests such as hepatitis B surface antigen, envelope antigen, and viral DNA. Clinicians can make a probable distinction between acute and chronic disease by considering the context of diagnosis–asymptomatic patients diagnosed after incidental discovery of elevated transaminases most likely have chronic disease whereas newly symptomatic patients with elevated transaminases likely have acute disease. This distinction is not entirely reliable, however, since new infections, hepatotoxins, cholelithiasis, and other unidentified factors can cause dramatic “flares” of chronic hepatitis B that resemble acute infection. Laboratory systems can only identify acute cases amongst patients that have positive tests for IgM to hepatitis B core antigen but this test is rarely ordered by clinicians investigating hepatitis.[13]

Analysis of data captured in electronic medical record systems and regional health information exchanges might be able to overcome the limitations of both clinician-initiated and electronic laboratory reporting of acute hepatitis B. Integration of multiple streams of electronic health data present in these systems such as current and prior diagnoses, prescriptions, and laboratory results may yield enough information to distinguish acute infection from chronic disease. We consequently sought to create and validate an algorithm to distinguish acute from chronic hepatitis B using codified electronic medical record data to facilitate automated public health surveillance.

## Methods

The clinical surveillance definition for acute hepatitis B published by the Centers for Disease Control and Prevention (CDC), shown in Table 1, was adapted to create two pilot electronic algorithms: 1) serum transaminases >5 times normal and positive IgM to hepatitis B core antigen, and 2) serum transaminases >5 times normal and a positive hepatitis B specific test (surface antigen, antibody to core antigen, or DNA).[11] The algorithms were refined by excluding all patients with prior positive laboratory tests for hepatitis B or an ICD9 code for chronic hepatitis B. We then tested the algorithms by applying them to comprehensive electronic medical record data from Harvard Vanguard Medical Associates from January 1990 through May 2006. Harvard Vanguard Medical Associates is a large, multispecialty, ambulatory medical practice based in Eastern Massachusetts with approximately 350,000 patients. The chart of each patient identified by the algorithms was reviewed by an infectious disease specialist to establish a diagnosis of acute versus chronic disease using the CDC definition as a reference standard.

**Table 1:** Centers for Disease Control and Prevention surveillance definition for acute hepatitis B.

**Clinical Criteria**	An acute illness with:
	a) discrete onset of symptoms **and**
	b) jaundice or elevated serum aminotransferase levels
**Laboratory Criteria**	IgM antibody to hepatitis B core antigen (anti-HBc) positive **OR**
	hepatitis B surface antigen (HBsAg) positive **AND**
	IgM antibody to hepatitis A negative

Acute hepatitis B was defined as the presence of a positive hepatitis B specific test (surface antigen or envelope antigen or DNA or antibody to hepatitis B core antigen) and one or both of the following: 1) an acute presentation of symptomatic disease consistent with hepatitis B (fever, nausea, vomiting, abdominal pain, fatigue, myalgias, jaundice, dark urine, and/or pale stool); and 2) prior or subsequent negative surface antigenemia without intervening hepatitis B therapy. Chronic hepatitis B was defined as a positive hepatitis B specific test in a patient who was asymptomatic or had a known history of hepatitis B by patient report or prior positive hepatitis B specific tests. Candidate algorithms were refined based on manual analysis of false positive cases identified by the algorithm.

The final algorithm was validated by applying it to an independent dataset of electronic medical record data gathered from Atrius Health between June 2006 and April 2008. Atrius Health is the product of a merger between Harvard Vanguard Medical Associates and four other ambulatory medical practices in Eastern Massachusetts. The combined practice serves over 600,000 patients at 35 clinical sites. The algorithm was applied to the Atrius Health dataset within the test environment of a novel electronic system designed to prospectively scan electronic medical record data to automatically identify and report notifiable diseases on a daily basis.[8], [14] We assessed completeness of case capture in the validation dataset by comparing its incidence-density of acute hepatitis B with the annual incidence density in the three most recent years of the derivation set. Recent annual incidence-densities were chosen over incidence-density of the full dataset because the incidence of hepatitis B has been dropping dramatically since universal vaccination was introduced in 1991.[11] We also validated the final algorithm against an external standard by searching state health department records for all cases of acute hepatitis B diagnosed between June 2006 and April 2008 to determine whether any cases independently reported by Atrius clinicians or laboratories were missed by the algorithm.

For comparison sake, we also estimated the positive predictive value of identifying acute hepatitis B purely from the presence of an ICD9 code for acute hepatitis B (070.30). We did so by selecting 50 patients at random from amongst all who were given this code within the past two years.

All candidate algorithms are presented in Table 2.

**Table 2:** Sensitivity and specificity of candidate algorithms to identify acute hepatitis B.

Algorithm		Algorithm Components	Total Patients	Positive Predictive Value	Sensitivity	Specificity
A	1 only	1. ICD9 for acute hepatitis B (070.30)	2564	0/50 (0%, 95% CI, 0–6%)*	***	***
B	(1and 2) or 3	1. ALT or AST≥5× normal OR ICD9 for jaundice AND	272	117/248 (47.2%, 95% CI, 41–53%)**	***	***
		2. Anti-HBc reactive within a 14 day period OR HBsAg positive within a 14 day period OR hepatitis B viral DNA positive within a 14 day period				
		3. Current HBsAg positive with prior HBsAg negative within preceding year				
C	(1and 2 and 3) or 4	1. ALT or AST≥5× normal OR ICD9 for jaundice AND	195	117/171 (68.4%, 95% CI, 61–75%) **	***	***
		2. Anti-HBc reactive within a 14 day period OR HBsAg positive within a 14 day period OR hepatitis B viral DNA positive within a 14 day period				
		3. No ICD9 code for chronic hepatitis B (070.32) in medical record OR prior positive HBsAg OR prior positive hepatitis B viral DNA				
		4. Current HBsAg positive with prior HBsAg negative within preceding year				
D	(1 and 2 and 3 and 4) or 5	1. ALT or AST≥5× normal OR ICD9 for jaundice AND	115	111/115 (96.5%, 95% CI, 93–100%)	111/117 (94.9%, 95% CI, 91–99%)	50/54 (92.6%, 95% CI, 86–100%)
		2. Anti-HBc reactive within a 14 day period OR HBsAg positive within a 14 day period OR hepatitis B viral DNA positive within a 14 day period				
		3. No ICD9 code for chronic hepatitis B (070.32) in medical record OR prior positive HBsAg OR prior positive hepatitis B viral DNA				
		4. ALT>1000				
		5. Current HBsAg positive with prior HBsAg negative within preceding year				
E	(1 and 2 and 3 and 4) or 5	1. ALT or AST≥5× normal OR ICD9 for jaundice AND	115	112/115 (97.4%, 95% CI, 94–100%)	112/113 (99.1%, 95% CI, 97–100%)	45/48 (93.8%, 95% CI, 87–100%)
		2. Anti-HBc reactive within a 14 day period OR HBsAg positive within a 14 day period OR hepatitis B viral DNA positive within a 14 day period				
		3. No ICD9 code for chronic hepatitis B (070.32) in medical record OR prior positive HBsAg OR prior positive hepatitis B viral DNA				
		4. Total bilirubin>1.5				
		5. Current HBsAg positive with prior HBsAg negative within preceding year				

ABBREVIATIONS: ICD9–International Classification of Disease, Ninth Revision, Clinical Modification; ALT–alanine aminotransferase; AST–aspartate aminotransferase; HBc–hepatitis B core antigen; HBsAg–hepatitis B surface antigen.

* random selection of 50 patients seen between 2006 and 2007.

** denominators lower than total number of patients due to missing data and exclusion of ambiguous cases.

*** sensitivity and specificity not available for algorithms A–C since the reference standard for sensitivity and specificity calculation derived from chart review of patients identified by algorithm C.

## Results

Analysis of electronic medical record data spanning 1990 through May 2006 yielded 11 patients with transaminases >5 times normal and positive IgM to hepatitis B core antigen. A second analysis for patients with transaminases >5 times normal and at least one specific hepatitis B test within a 14 day period yielded 272 cases of possible acute hepatitis B, including all 11 patients with positive IgM to core antigen. Exclusion of patients with an ICD9 code for chronic hepatitis B or prior positive laboratory tests for hepatitis B reduced the number of cases to 195. Full text charts could not be located for 13 patients. Charts on the remaining 182 patients were reviewed by an infectious disease physician. Of these, 117 fulfilled criteria for acute hepatitis B and 54 for chronic hepatitis B. A confident diagnosis could not be rendered in the remaining 11 cases. These were patients who lacked clear documentation of their presenting symptoms, who presented acutely with atypical symptoms (e.g. isolated epigastric burning responsive to proton pump inhibitors), or had potential alternative explanations for acute hepatitis (e.g. recent initiation of hepatotoxic medications).

The accuracy of each candidate algorithm is presented in Table 2. Simple presence of an ICD9 for acute hepatitis B without regard to any other criteria had a positive predictive value of 0% (0/50 cases, 95% confidence interval 0–6%). By contrast, CDC criteria (elevated transaminases and a positive hepatitis B specific test–algorithm B) yielded a positive predictive value of 47.2% (117/248, 95% confidence interval 41–53%). Exclusion of patients with prior hepatitis B positive tests or ICD9 codes for chronic hepatitis B (algorithm C) raised the positive predictive value to 68.4% (117/171, 95% confidence interval 61–75%). The addition of a requirement for ALT>1000 (algorithm D) raised the positive predictive value to 96.5% (111/115, 95% confidence interval 93–100%) with sensitivity 95% (111/117, 95% confidence interval 91–99%) and specificity 93% (50/54, 95% confidence interval 86–100%). Algorithm E substituted total bilirubin>1.5 rather than ALT>1000. This yielded a positive predictive value of 97.4% (112/115, 95% confidence interval 94–100%) with sensitivity 99% (112/113, 95% confidence interval 97–100%) and specificity 94% (45/48, 95% confidence interval 87–100%).

Algorithm E was subsequently applied to prospectively collected electronic medical record data from over 600,000 patients seen in Atrius Health between June 2006 and April 2008. During this period, 2684 positive hepatitis B specific tests were obtained for 601 patients. Of these, 8 were flagged as acute hepatitis B by algorithm E. Chart review confirmed all 8 to be true positive cases (100% positive predictive value).

**Figure 1:**
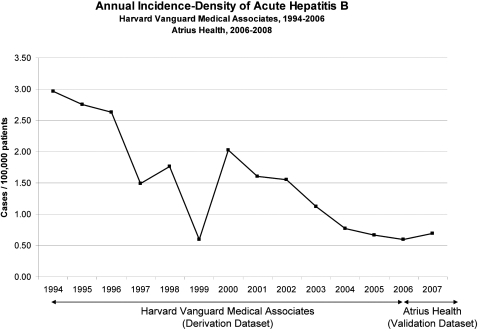
Annual incidence-density of acute hepatitis B in the derivation dataset (Harvard Vanguard Medical Associates, January 1994–May 2006) and in the validation dataset (Atrius Health, June 2006–April 2008).

The incidence-density of acute hepatitis B in the validation set was 0.70 cases/100,000 patients. For comparison sake, the annual incidence density in derivation set in the years 2004, 2005, and 2006 was 0.77, 0.67, and 0.59 cases/100,000 patients respectively (Figure 1).

State health department records of acute hepatitis B cases diagnosed during the validation period were searched for patients with acute hepatitis B independently reported by laboratories and Atrius Health clinicians. Of the 8 cases found by the algorithm, 4 were already known to the state health department from spontaneous reporting but only 1 of those 4 cases was labelled as acute infection. The other 3 were recorded as hepatitis B without comment on whether acute or chronic. There were no Atrius Health patients with acute hepatitis B known to the state health department that were missed by the algorithm.

## Discussion

Algorithms applied to electronic medical record data can accurately identify cases of acute hepatitis B. The best electronic algorithm achieved a sensitivity of 99% and specificity of 94% for acute hepatitis B. When applied to two years of prospective electronic medical record data, the algorithm found 8 true cases including 4 cases that clinicians and laboratories had failed to report to the health department, and 3 cases reported to the health department as hepatitis B alone without indication of whether acute or chronic. There were no false positive cases and no known cases missed.

The high accuracy of the final algorithm was achieved by integrating multiple streams of data from the electronic medical record including current biochemical tests, the results of prior hepatitis B testing, and ICD9 coding. Of note, 2 acute biochemical findings appear helpful to identify acute infections: peak ALT>1000 and total bilirubin>1.5.

The single case of confirmed acute hepatitis B in our cohort without a total bilirubin>1.5 may have been an artefact of timing of lab measurements. This patient only had bilirubin measured at the time of initial presentation. The patient's transaminases continued to rise in subsequent days but his bilirubin was not measured again. Since bilirubin elevation is known to lag slightly behind transaminase elevation, the patient might have met algorithm criteria for acute infection if bilirubin been measured again on a subsequent visit.

Neither ALT>1000 nor total bilirubin>1.5 criteria are 100% specific. Four patients with chronic hepatitis B presented with ALT >1000 and 3 patients with chronic infection had total bilirubin >1.5. One patient with chronic hepatitis B who presented with an ALT of 1086 was diagnosed with cholecystitis. There were no clear precipitants identified for the unusually high ALT values seen in the other three patients with underlying chronic hepatitis B. Sources of hyperbilirubinemia in chronic hepatitis B patients included cholecystitis and end-stage cirrhosis.

These false positives are consistent with previous studies in which patients with flares of chronic hepatitis B occasionally present with very high transaminases and bilirubin. Davis and Hoofnagle, for example, prospectively followed 150 patients with chronic hepatitis B and found that two developed clinical jaundice from flares of their hepatitis B.[15] Our algorithm is designed to minimize these sources of false positives by excluding patients with prior positive hepatitis B tests or an ICD9 code for chronic infection in their electronic medical records. These exclusion criteria combined with the rarity of cholestasis in severe flares of chronic hepatitis B likely account for the high specificity of our algorithm despite case reports of jaundice in flares of chronic infection.

It is unlikely that the physician chart reviewer's subjective judgment of acute versus chronic disease influenced the relative performance of the algorithms. Serial hepatitis B surface antigen tests were available for 82% of patients; the patterns of change in surface antigenemia over time confirmed the physician reviewer's clinical impression in all cases in which serial tests were available. These confirmatory changes in surface antigenemia decrease the likelihood that acute cases of anicteric disease were misclassified as chronic infections.

Previous studies suggest that some cases of acute hepatitis B are clinically silent.[16], [17] These patients were likely missed by this analysis since by definition it was limited to patients who presented for clinical evaluation. Our algorithms do incorporate a strategy for seeking clinically silent acute cases of disease (serial change in hepatitis B surface antigen from negative to positive in a patient without known prior infection) but this strategy is still contingent upon patients with silent disease presenting for clinical care and eliciting sufficient clinical suspicion to prompt serial surface antigen testing. These are admittedly rare circumstances.

The poor positive predictive value of ICD9 code 070.30 for acute hepatitis B (0%, 95% confidence interval 0–6%) is likely an artefact of the text description given to this code in our practice's electronic medical record. It is labelled as “hepatitis B” alone rather than “acute hepatitis B” and hence is commonly used by clinicians for asymptomatic patients found to have evidence of remote exposure to hepatitis B or ongoing chronic disease despite the presence of a specific alternative code for chronic disease. The poor performance of ICD9 codes for hepatitis surveillance is consistent with previous work and underscores the poor accuracy of disease surveillance using ICD9 codes alone.[10]

Similarly, the small number of cases of acute disease detected by screening for positive IgM to hepatitis B core antigen reveals the limitation of population surveillance for acute disease using this test alone. The poor sensitivity of IgM to core antigen for population-level surveillance is a consequence of the test rarely being ordered. In our series of 195 patients presenting with elevated transaminases and a positive hepatitis B specific test, only 20 patients went on to have IgM to core antigen assayed.

Analysis of the distribution of other positive hepatitis B specific tests relative to the number of patients ultimately found to have acute hepatitis B is a further window into the benefit of comprehensive electronic medical record data for notifiable disease surveillance relative to conventional laboratory-based reporting systems. Laboratory-based reporting systems would have generated 2648 reports of patients with hepatitis B without flagging the eight acute cases from the many more chronic cases (Table 3). By contrast, an algorithm leveraging diverse streams of electronic medical record data reliably identified the handful of acute cases within this large pool of positive tests.

**Table 3:** Total number of positive tests, patients, and acute cases of hepatitis B in Atrius Health (June 2006 through April 2008).

Total number positive hepatitis B specific tests (HBsAg, HBeAg, HBV DNA)	2,648
Total number of patients with positive hepatitis B specific tests	601
Total number of patients with acute hepatitis B	8

ABBREVIATIONS: HBsAg–hepatitis B surface antigen; HBeAg–hepatitis B envelope antigen; HBV DNA–hepatitis B viral DNA.

A potential limitation of this work is the small size of the validation dataset relative to the derivation set. Nonetheless, disparate lines of evidence suggest that the validation is accurate. In and of itself, the validation set is large, covering 1.2 million patient-years. All cases found in the validation set were true positives, mirroring the high positive predictive value of the algorithm in the derivation set. The incidence-density of acute hepatitis B in the validation set closely matched the incidence-density in the final years of the derivation set. Finally, comparison of case capture in the validation set with the state health department's database of independently reported cases of acute hepatitis B failed to reveal any cases missed by the algorithm.

This work shows that it is possible to accurately identify acute hepatitis B from electronic medical record data. The final algorithm described in this work is now being used for live, prospective surveillance within Atrius Health–the last 3 of the 8 acute cases described in this dataset were prospectively detected. The performance of the acute hepatitis B algorithm suggests that it is feasible to overcome some of the limitations of clinician-initiated and electronic laboratory based reporting of notifiable diseases by identifying complex diseases from electronic medical records. Integration of algorithms such as the one developed here into live disease detection and reporting systems that analyze real-time electronic health data promises to improve the quality, completeness, and timeliness of public health surveillance.
